# Supplementary material for: Heart rate and heart rate variability in horses undergoing hot and cold shoeing
Source: PLoS One. 2024 Jun 6;19(6):e0305031. doi: 10.1371/journal.pone.0305031 (PMC11156413; doi:10.1371/journal.pone.0305031)
Supplement: S1 Fig — A trend toward an increase in the high-frequency domain is observed at 30–90 min post-shoeing in horses undergoing hot shoeing. (DOCX) [file pone.0305031.s001.docx]

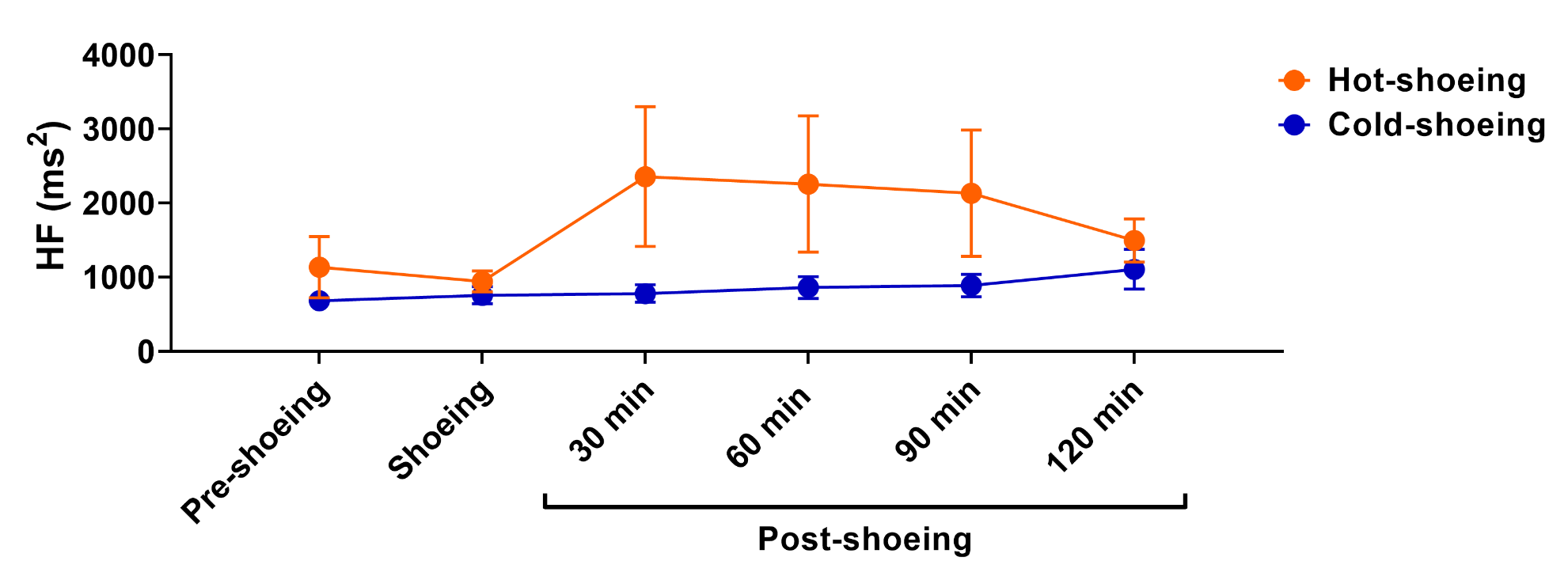


**Fig S1** The high-frequency domain of heart rate variability in horses receiving the hot and cold shoeing protocol. A trend to increase in the high-frequency domain is observed at 30–90 min post-shoeing in horses receiving the hot shoeing protocol.
