# Supplementary material for: Heart rate and heart rate variability in horses undergoing hot and cold shoeing
Source: PLoS One. 2024 Jun 6;19(6):e0305031. doi: 10.1371/journal.pone.0305031 (PMC11156413; doi:10.1371/journal.pone.0305031)
Supplement: S1 Table — (DOCX) [file pone.0305031.s002.docx]

**Table S1** Horses with different disciplines receiving hot-shoeing or cold-shoeing methods

| **Horse no.** | **Disciplines** | | | | | | **Hot-shoeing** | **Cold-shoeing** |
| --- | --- | --- | --- | --- | --- | --- | --- | --- |
|  | **Dressage** | **Jumping** | **Eventing** | **Polo** | **School riding** | **Leisure** |  |  |
| 1 | **/** |  |  |  |  |  | **/** |  |
| 2 | **/** |  |  |  |  |  | **/** |  |
| 3 |  | **/** |  |  |  |  | **/** |  |
| 4 | **/** |  |  |  |  |  | **/** |  |
| 5 |  | **/** |  |  |  |  | **/** |  |
| 6 |  | **/** |  |  |  |  | **/** |  |
| 7 |  | **/** |  |  |  |  | **/** |  |
| 8 |  | **/** |  |  |  |  | **/** |  |
| 9 |  | **/** |  |  |  |  | **/** |  |
| 10 |  | **/** |  |  |  |  | **/** |  |
| 11 |  | **/** |  |  |  |  | **/** |  |
| 12 |  | **/** |  |  |  |  | **/** |  |
| 13 |  | **/** |  |  |  |  | **/** |  |
| 14 |  |  |  |  | **/** |  | **/** |  |
| 15 |  |  |  |  | **/** |  | **/** |  |
| 16 |  |  |  |  |  | **/** | **/** |  |
| 17 |  |  |  |  | **/** |  | **/** |  |
| 18 |  |  |  |  | **/** |  | **/** |  |
| 19 |  |  |  |  |  | **/** | **/** |  |
| 20 |  |  |  |  |  | **/** | **/** |  |
| 21 |  |  |  |  |  | **/** | **/** |  |
| 22 |  | **/** |  |  |  |  | **/** |  |
| 23 |  |  |  |  |  | **/** | **/** |  |
| 24 |  | **/** |  |  |  |  | **/** |  |
| 25 |  |  |  | **/** |  |  | **/** |  |
| 26 |  |  |  | **/** |  |  | **/** |  |
| 27 |  |  |  | **/** |  |  |  | **/** |
| 28 |  |  |  | **/** |  |  |  | **/** |
| 29 |  |  |  | **/** |  |  |  | **/** |
| 30 |  |  |  | **/** |  |  |  | **/** |
| 31 |  |  |  | **/** |  |  |  | **/** |
| 32 |  |  |  | **/** |  |  |  | **/** |
| 33 |  |  |  | **/** |  |  |  | **/** |
| 34 |  |  |  | **/** |  |  |  | **/** |
| 35 |  |  |  | **/** |  |  |  | **/** |
| 36 |  |  |  | **/** |  |  |  | **/** |
| 37 |  |  |  | **/** |  |  |  | **/** |
| 38 |  |  |  | **/** |  |  |  | **/** |
| 39 |  |  |  | **/** |  |  |  | **/** |
| 40 |  |  |  | **/** |  |  |  | **/** |
| 41 |  |  |  | **/** |  |  |  | **/** |
| 42 |  |  |  | **/** |  |  |  | **/** |
| 43 |  |  |  | **/** |  |  |  | **/** |
| 44 |  |  |  | **/** |  |  |  | **/** |
| 45 |  |  |  | **/** |  |  |  | **/** |
| 46 |  |  |  | **/** |  |  |  | **/** |
| 47 |  |  |  | **/** |  |  |  | **/** |
| 48 |  |  | **/** |  |  |  |  | **/** |
| 49 |  | **/** |  |  |  |  |  | **/** |
| 50 |  | **/** |  |  |  |  |  | **/** |
| 51 |  |  |  |  |  | **/** |  | **/** |
