# Supplementary material for: Heart rate and heart rate variability in horses undergoing hot and cold shoeing
Source: PLoS One. 2024 Jun 6;19(6):e0305031. doi: 10.1371/journal.pone.0305031 (PMC11156413; doi:10.1371/journal.pone.0305031)
Supplement: Attachment — Submitted filename: PONE feedback author.docx [file pone.0305031.s003.docx]

- Generally this article is a nice study on the effects of shoeing on stress in horses which may be relevant for the welfare of horses. However, it is unclear what definition is used of stress and what exactly is investigated. Therefore, the study does need some work and may benefit from clarification. Hopefully the comments below will aid in this process.
- 31: during which time period more sympathovagal activity?
- 32/33: Increases or decreases in sympathovagal activity do not necessarily mean differences between horses that are hot-shoed or cold-shoed. Can you draw the conclusion from you results that hot-shoeing provides more stress than cold-shoeing? Perhaps the results are better reflected by the sentence: hot-shoed horses experience stress whereas cold-shoed horses do not show the same response?
- No differences between pre and during shoeing?
- 38: Does HRV only reflects short term fluctuations, or does that depend on the type of parameter you use?
- 39: does the fluctuation accompany oscillatory influences or does it reflect them?
- 43: what does the author use as indication of stress, is this physiological stress or mental stress? The literature referred to is mostly directed at physiological stress. If the author also would like to include psychological stress please include more literature on this.
- 64: is referring to human literature here relevant? If there is quite a substantial amount of literature on horses why would you include human literature?
- 67: perhaps this Alinea (no. 3) should be moved to the start the article, as it become clear than that it covers the procedures of horseshoeing and why it is relevant to investigate this further
- 71: “wildly”> I think the author means “widely”?
- 73: what does the author mean by “despite a positive outcome” that generally shoeing is better for the hoof condition? In the second part of the sentence: please be more specific about which shoeing protocols may negatively impact the hooves and why is this relevant for your article?
- 78: The author indicates the gap in literature but not why is it relevant to investigate differences between shoeing methods. Are there indications that one is better for the welfare of the horse or is less stressful than the other? Please spend some more explanation on this topic so that it becomes more clear why your study is relevant for the field. I assume the author is interested in psychological stress of shoeing methods and that HRV parameters are a good way to measure this but the introduction does not clearly explain this.
- 85: this study did not include young horses, is that a deliberate choice? Were all these horse familiar with both shoeing methods previously before they were included in the study? Since the horses were recruited form equestrian clubs I assume they were habituated to wearing a girth and/or did you habituate them to wearing the polar strap?
- 93: Were these horses recruited and housed at the same stable or did you measure them at their own location? If they are in their own location and treated by their own familiar farrier, how did you control for differences in farriers?
- 105: did you soke the belt or did you wet the fur of the horses? Did you not use a lubricant gel and how about the condition of the fur did you shave them or not? All these factors do matter for how well your measurements are. What is the percentage of beat correction and did you use a correction factor?
- 110: what do you mean by the “calm station” ?
- 111: do you mean hot or cold shoeing methods by ‘protocol’ or did the farriers receive a protocol to standardize the treatment as much as possible?
- 133: why did you use polar equipment and not the gold standard ECG measurement? ECG measurement allows you to do the corrections manually and is more accurate in HRV measurement. If there is a reason why polar equipment is preferred for this setup explain this in the introduction.
- 149: the standard settings in Kubios are frequency bands are used for human data, why did you choose not to change the frequency bands for the horse data?
- 162: please explain if your dataset has met the assumptions of a two-way ANOVA, for example were all your HRV parameters normally distributed?
- 191: I would have expected the shoeing procedure to be stressful but this study did not find differences in HR and RR when comparing pre and during time periods. This would actually indicate that they are both not stressful procedures. This also makes me wonder where the pre time period was measured, is this done in their stable or in the shoeing box? If the pre measurement is taken in the ‘calm box’ this might make the horses anticipate to the shoeing before the actual treatment starts and might cause psychological stress. This may also explain why you do not find differences between pre and during periods. If the post period measurements are done within their own stable this might also explain the increase in HRV. I also wonder how controlled the post time periods are. Could there be feeding procedures taking place which might influence HRV parameters for example?
- Results section in general: is there added value in providing all these parameters in both time, frequency and non-linear variables? Why for example not only provide the SD1/SD2 ratio for the non-linear variables.
- 285: the author explains clearly why only HR measurements are not enough, however no results are discussed against literature references. Therefore I wonder if this Alinea should not be placed to the introduction section?
- 296: see above, the same accounts for this Alinea, the explanation why you used a HRM could also be part of the introduction or material& method section.
- 310: ‘more precise’ compared to what?
- 326: The author suggests that horses experiences stress relief in the hooves following hot shoeing, however it could also be that during the pre and during time period the horses were anticipating and stressed about the shoeing process, which decreased during the 30 min. time period. Not so much indicating physiological stress relief but reduction in psychological stress. Please substantiate this statement with literature and also consider other possible explanations.
- 338: There are also indications in literature that you should adapt the frequency band thresholds for horses, please substantiate your choice further and discuss this in more depth.
- 348: pleas rephrase this line and replace the word excellent, for example by: “These results indicate a larger contribution of sympathovagal activity…..”
- 378-379: how an this increased vagal activity at 30 min. and increased sympathovagal activity at 60-120 min. be explained, did you control for activity/locomotion behaviour for example? It could mean that the horses were less active at 30 min. showing more resting behaviour and more active at 60 min. but is this due to the shoeing method or could there be differences in regime at the several housing locations for example feeding practices?
- 379-382: during what time period did you observe no change in SD2/SD1 and HF/LF ratio? “….supported the observance of the integral generation of…..” this a bit vagely formulated. The paragraph is well explained in general and shows nice depth in the discussion. However this last sentence needs some work.
- 386-388: I would have expected differences between pre and during shoeing methods, how can you explain that you did not find this? Did you “assume” or do you mean “hypothesize”, and would you expect the different shoeing methods to show no differences?
- 392: remove the word excellent
- 393-394: previously you stated that you expected no differences but this sentence contradicts it since here you state that you expect horses to show lower stress levels. The phrase “be lower stress” is not correctly formulated, please replace it with: show lower stress levels.
- 397: this study does not investigate the reliability of the measurement, perhaps replace it with the usefulness/applicability? And what do you intend to measure: psychological stress or physiological stress? Since later in the paragraph you mention that shoeing can affect the horse’s gait so perhaps the time effect is not so much due to stress but to locomotion?
- 402: ‘…..continued study ….is required in athletic horses’, you only studied HRV variables so in my opinion the author cannot make inferences from this on locomotion and exercise as a parameter. Although it might have been a good idea to also take locomotion into account for this study as a parameter.
- 404: limitations of the study do not discuss the fact that you might have a bias in your results as the hot shoeing procedures are applied to jumping horses and horses from riding schools, whereas cold shoeing was applied to polo horses, this might affect your results due to differences in training, body condition, character etc.
- 405: stress does not lead to errors in HRV but psychological stress affects your HRV parameters just as much as physiological stress. Did you record which horses were treated with an unfamiliar type of horseshoeing and check whether this factor had an effect?
- 407: the length of the time frame might also affect some of your HRV parameters, did you control for this and were all time periods of the same duration?
- 413: ‘although the horse applying….”sentence is not correctly formulated and therefore unclear
- 416: I wonder what the definition of stress is in this study, do you mean psychological stress or physiological stress?
