# Supplementary material for: Heart rate and heart rate variability in horses undergoing hot and cold shoeing
Source: PLoS One. 2024 Jun 6;19(6):e0305031. doi: 10.1371/journal.pone.0305031 (PMC11156413; doi:10.1371/journal.pone.0305031)
Supplement: Attachment — Submitted filename: PONE feedback author.docx [file pone.0305031.s004.docx]

**The manuscript entitled** “Heart rate and heart rate variability in horses receiving hot shoeing and cold shoeing protocols”

**Dear Editor,**

I’d appreciate your dedicated time in reviewing our manuscript and giving valuable comments to improve the context of this manuscript. We’ve addressed all points raised by the reviewers, and the track changes within the text noticed this modification.

**Editor’s comments**

The presented manuscript relates to a study on the effect of two shoeing methods on HRV parameters in horses. The aims of the study are of interest however it is not clear whether the study intends to investigate psychological stress and/or physiological stress. The subject of the submitted draft is within the scope of PLOS ONE. The manuscript is well written. The introduction is well composed and introduces well the question. The Materials and Methods section could be more detailled to present the conditions in which the measurements were performed. Although the used statistical analysis seems appropriate, more explanation on whether the requirements for performing this two-way ANOVA were met. The Results section could be more synthesized in the text. The Discussion section is well written and the author did discuss well the results of the study, including the limitations.

Response to the editor:

As per the editor’s question about the objective of this study, this study aims to investigate the physiological stress (autonomic response) in response to the usual shoeing protocols in horses. Generally, conventional shoeing methods by professional farriers usually offer horses comfort after shoeing. The difference in autonomic response in horses receiving shoeing should be more or less comfortable rather than higher or lower stress following shoeing protocol. After careful revision, we decided to use the term “comfort” instead of “stress” to reflect the outcome of shoeing protocols as it delivers a more explicit message from this study. We’ve modified the main text over this issue on page 1, line30-32, and page 20, lines 417 and 421.

Additionally, we haven’t used the track change method in this revision to demonstrate where the text has been corrected. As there are a lot of corrections and modifications within the main text, we decided to get the full revised manuscript grammatically corrected by the Cambridge proofreading service (the proofreading certificate was attached to the resubmission system). So, the corrections and modifications according to the reviewers’ comments were highlighted in green.

**Reviewers’ comments**

- Generally this article is a nice study on the effects of shoeing on stress in horses which may be relevant for the welfare of horses. However, it is unclear what definition is used of stress and what exactly is investigated. Therefore, the study does need some work and may benefit from clarification. Hopefully the comments below will aid in this process.

Response to the reviewer:

We’d like to add more information regarding Heart rate variability (HRV) modulation and stress response to stimulus. HRV has frequently been used for indicating stress response in horses during exercise (https://doi.org/10.3389/fphys.2016.00155), with pain (https://doi.org/10.1016/j.jveb.2017.09.002), and other stressors such as transport (https://doi.org/10.1016/j.yhbeh.2009.11.003).. An increase in HRV, indicated by increased several HRV variables, reflects parasympathetic dominance, more excellent adaptive capability, and lower stress in response to challenges. Regarding this study, we found that several HRV variables increased significantly during the hot shoeing protocol, lasting until 120 minutes post-shoeing. Moreover, horses receiving hot shoeing showed higher HRV than those with cold shoeing throughout the 120-minute post-hot shoeing period. This increased HRV reflects more body adaptability to cope with challenges. Therefore, those horses with hot shoeing are more comfortable than those with cold shoeing periods after shoeing. The sentences to deliver this message were modified on page 2, lines 30-32, and page 20, lines 417-421.

More importantly, we have decided to use “more comfort” instead of “lower stress” following increased or higher HRV in horses receiving hot shoeing to avoid confusion and misconduct in the messages. That’s because the appropriate shoeing method typically causes the horse to be comfortable after shoeing. The increased HRV or higher HRV should mean more comfort rather than lower stress.

- 31: during which time period more sympathovagal activity?

Response to the reviewer:

We do apologize for giving the unsuitable word and misleading the reader with this term. In this sentence, we’ve changed to use the parasympathetic activity instead of the sympathovagal activity. This study found that the SDNN, TINN, HRV triangular index, LF power spectrum and SD2 increased during 30-120 minutes. So, the more sympathovagal activity in this context was found throughout 120 minutes post-shoeing in horses with hot shoes. We

- 32/33: Increases or decreases in sympathovagal activity do not necessarily mean differences between horses that are hot-shoed or cold-shoed. Can you draw the conclusion from you results that hot-shoeing provides more stress than cold-shoeing? Perhaps the results are better reflected by the sentence: hot-shoed horses experience stress whereas cold-shoed horses do not show the same response?

Response to the reviewer:

As explained above, appropriate conventional shoeing methods by professional farriers offer horses comfort, indicated by increased HRV in horses after shoeing. Higher HRV in horses after hot shoeing should be declared as more comfort rather than lower stress. So, “comfort” was used instead of “stress” in this context. We’d like to explain a little bit regarding the conclusion of this study. In the present study, an increase in mean RR, pNN50, TINN, HRV, and triangular index, for example, was observed in horses receiving both shoeing protocols, supposing that horses derive comfort after shoeing compared to before shoeing. However, the higher HRV after shoeing in horses with hot shoeing compared to cold shoeing. This result reflects an excellent sympathovagal action leading to higher HRV and mirroring more comfort in hot-shoed horses than cold-shoed horses after shoeing. This study concluded that horses derive more comfort after applying hot shoeing than cold shoeing.

- No differences between pre and during shoeing?

Response to the reviewer:

There was no statistical difference between pre- and during shoeing in horses receiving both types of shoeing protocol. The difference was majorly noticed after shoeing.

- 38: Does HRV only reflects short term fluctuations, or does that depend on the type of parameter you use?

Response to the reviewer:

We do apologize for giving the reviewers an unclear message over this issue. The HRV variables can reflect both short-term (RMSSD, NN50, pNN50, HF power spectrum and SD1 nonlinear results) and long-term fluctuation (mean RR, SDNN, TINN, HRV triangular index, LF power spectrum, SD2 nonlinear results) of heartbeats. To avoid misconducting this message, we’ve modified the sentence of the introduction part to “Heart rate variability (HRV) is the time difference between beat-to-beat (RR) intervals during the cardiac cycle” on page 3, lines 53-54.

- 39: does the fluctuation accompany oscillatory influences or does it reflect them?

Response to the reviewer:

We’d like to explain a little bit regarding the HRV modulation. The contraction and relaxation of the heart muscle in the cardiac cycle accompany the rhythmic impulse of the autonomic nervous system (ANS), including sympathetic and parasympathetic components, acting on the heart’s sinoatrial node. So, the variation of the time interval between heartbeats is under the oscillatory influences of both ANS components as well as reflects the synchronous activities of both parts.

- 43: what does the author use as indication of stress, is this physiological stress or mental stress? The literature referred to is mostly directed at physiological stress. If the author also would like to include psychological stress please include more literature on this.

Response to the reviewer:

We do apologize again for giving the reviewers an unclear definition of stress. It was physiological stress, as per the reviewer’s mention. We’ve modified it accordingly on page 3, lines 59-60.

- 64: is referring to human literature here relevant? If there is quite a substantial amount of literature on horses why would you include human literature?

Response to the reviewer:

Thank you for your question. In this connection, we agree with the reviewer there is a lot of literature regarding HRV in horses. However, we just want to address the use of the HRV parameter in humans in a few sentences because we’d like to convince the readers of the worldwide reliability of using the HRV parameter to indicate stress response in horses and humans. However, after careful consideration, we removed it from the text and left only the HRV modulation in horses in this manuscript.

- 67: perhaps this Alinea (no. 3) should be moved to the start the article, as it become clear than that it covers the procedures of horseshoeing and why it is relevant to investigate this further

Response to the reviewer:

Thank you for your suggestion. We’ve moved as well as modified the text of the shoeing protocol description to the first paragraph of the introduction part on page 3, lines 36-45.

- 71: “wildly”> I think the author means “widely”?

Response to the reviewer:

Thank you for your suggestion. We’ve revised it accordingly on page 3, line 39.

- 73: what does the author mean by “despite a positive outcome” that generally shoeing is better for the hoof condition? In the second part of the sentence: please be more specific about which shoeing protocols may negatively impact the hooves and why is this relevant for your article?

Response to the reviewer:

Thank you for your suggestion. We’ve modified the content regarding the shoeing method description on page 3, lines 36-45. The context you mentioned is removed accordingly.

- 78: The author indicates the gap in literature but not why is it relevant to investigate differences between shoeing methods. Are there indications that one is better for the welfare of the horse or is less stressful than the other? Please spend some more explanation on this topic so that it becomes more clear why your study is relevant for the field. I assume the author is interested in psychological stress of shoeing methods and that HRV parameters are a good way to measure this but the introduction does not clearly explain this.

Response to the reviewer:

We do apologize for giving the reviewers unclear rationale in this study. We conducted this study because farriers and practitioners believe that hot shoeing provides horses with more benefits due to a subjective evaluation as it improves juxtaposition between the hoof wall and metal shoe, and horseshoes can be fitted on hooves more accurately following the hot shoeing method. However, there is no report on objective evaluation indicating whether or not horses get more benefit from hot shoeing. In this connection, the HRV was subsequently selected to quantitatively determine those benefits by comparing HRV metrics between horses receiving hot and cold shoeing protocols. Unfortunately, no report compares HRV variables between horses receiving hot and cold shoeing protocols. This rationale leads us to conduct this research, objectively evaluating the benefits of horses fitting with hot shoes. We’ve modified the introduction part of the main text to explain the study’s rationale, the gap in the literature on page 3, lines 44-45, and the objective of this study on page 5, lines 91-95.

- 85: this study did not include young horses, is that a deliberate choice? Were all these horse familiar with both shoeing methods previously before they were included in the study? Since the horses were recruited form equestrian clubs I assume they were habituated to wearing a girth and/or did you habituate them to wearing the polar strap?

Response to the reviewer

Since some young horses are alert and spooky during the shoeing protocol, the HRV modulation may not solely result from the effect of shoeing methods. For this reason, we decided to recruit only well-educated adult horses, easy to restrain and get accustomed to the shoeing protocol, particularly shoeing by their familiar farriers. This subject recruitment was expected to significantly reduce confounding factors and produce more accurate study results. Regarding wearing the polar strap, all horses are used to getting this strap as it is like a belt of saddle, but much smaller, fastened around the chest. So, no need to habituate them to wearing the polar strap.

- 93: Were these horses recruited and housed at the same stable or did you measure them at their own location? If they are in their own location and treated by their own familiar farrier, how did you control for differences in farriers?

Response to the reviewer

All horses were housed and shod in their location and treated by their familiar farrier to get rid of any confounding factors such as fear, anxiety, and other aspects that can interfere with the HRV modulation other than shoeing protocols. In this study, approximately 50 horses were mainly shod by four professional farriers who perform shoeing according to the universal concept of shoeing methods such as cutting the hoof, rasping, and nailing. Typically, there is no difference in the shoeing concept between farriers as this is not the specific therapeutic shoeing but conventional shoeing methods for healthy horses that have been adopted by farriers worldwide. A little detail of shoeing practice is observed between them; for example, some start doing the hindlegs before the front legs with respect to the horses' characteristics, etc. By doing this, the autonomic response indicated by HRV modulation can primarily reflect the impact of the shoeing protocol in this study.

- 105: did you soke the belt or did you wet the fur of the horses? Did you not use a lubricant gel and how about the condition of the fur did you shave them or not? All these factors do matter for how well your measurements are. What is the percentage of beat correction and did you use a correction factor?

Response to the reviewer

Before fastening the strap, the belt was soaked in the water, and the area where the strap was around the chest was also wet. In fact, horses in Thailand have relatively short hair and are often wet with sweat due to the hot and humid climate. This condition transmits a sound signal between the sensor and the Polar sports watch with no need for hair shaving and the lubricant gel on the Polar strap throughout the study period. This study analyzed RR interval data from the Polar HR monitoring device for HRV variables using the premium version of HRV analysis software (Kubios HRV Scientific; <https://www.kubios.com/hrv-scientific/>). This premium program (subscription program) provides artifact correction algorithms that can automatically detect misaligned and ectopic beats such as premature ventricular contractions (PVC) or other arrhythmias. This automatic correction is more accurate than manual correction in the Kubios standard version (<https://doi.org/10.1080/03091902.2019.1640306>). Therefore, the program automatically corrected the misaligned and ectopic beats, which we set to a medium correction level. The beat correction is allowed not greater than 5%; however, we found that the ectopic beats corrected in this study were only0.6-1.4%, according to the Kubios HRV Scientific program.

- 110: what do you mean by the “calm station” ?

Response to the reviewer

We mean that horses were shod in the farrier shop without loud, horrible, or unfamiliar noise during shoeing. This arrangement aims to reduce the confounding factors causing the HRV modulation other than the effect of shoeing protocols.

- 111: do you mean hot or cold shoeing methods by ‘protocol’ or did the farriers receive a protocol to standardize the treatment as much as possible?

Response to the reviewer

The farrier performed hot and cold shoeing by protocol. As stated above, horses in this study were shod by professional farriers who perform shoeing according to the universal concept of shoeing methods such as cutting the hoof, rasping, and nailing. Typically, there is no difference in the shoeing concept between farriers as this is not the specific therapeutic shoeing but conventional shoeing methods for healthy horses that farriers have adopted worldwide.

- 133: why did you use polar equipment and not the gold standard ECG measurement? ECG measurement allows you to do the corrections manually and is more accurate in HRV measurement. If there is a reason why polar equipment is preferred for this setup explain this in the introduction.

Response to the reviewer

We agree with the reviewer that the ECG measurement, like the Holter device, is the goal standard for measuring RR interval for HRV derivation. However, the explanation in this topic has been placed in the discussion part. Due to the fact that we’d like to explain the intention to use the latest version of the Polar HR monitoring setting (polar belt, H10 sensor, and Vantage 2 polar sports watch) along with the latest version of the Kubios program that provides autonomic correction (noise and artifact correction that are provided only in the premium program). So, we prefer the leave the information regarding the use of Polar HRM in the discussion part. The explanation in this section is expected to convince the reader that we took the appropriate instrument and computer software to produce the reliable result of this study.

149: the standard settings in Kubios are frequency bands are used for human data, why did you choose not to change the frequency bands for the horse data?

Response to the reviewer

As per a report from Diana Stuck et al. (2015) (<https://doi.org/10.1016/j.applanim.2015.02.007>), several threshold settings are used for spectral analysis. There are a lot of studies using the frequency threshold for horses. However, Gehrke, E.K., Baldwin, A. and Schiltz, P.M. (2011) conducted research in horses adopting frequency threshold for analyzing spectral power in humans (<https://doi.org/10.1016/j.jevs.2010.12.007>). In addition, another study from our team has implemented the human frequency threshold for frequency domain analysis (<https://doi.org/10.1016/j.jevs.2023.104934>). In our opinion, applying the human frequency threshold is acceptable as long as there is no quantitative comparison with other HRV studies but within the current investigation.

- 162: please explain if your dataset has met the assumptions of a two-way ANOVA, for example were all your HRV parameters normally distributed?

Response to the reviewer

In this study, we haven’t tested the normal distribution of the data for Two-way ANOVA analysis. In fact, the dependent variable (residuals) should be approximately normally distributed for each cell of the design. The normality assumption is necessary for statistical significance testing using a two-way ANOVA. However, the two-way ANOVA is considered “robust” to violations of normality. This means that some violations of this assumption can be tolerated, and the test will still provide valid results. Furthermore, as sample size increases, the distribution can be pretty non-normal, but the two-way ANOVA can still provide valid results (<https://www.amstatisticalconsulting.com/two-way-anova/>). We guess that’s why there is no algorithm to test the normality of the data for the Two-way ANOVA statistical analysis in the latest version of GraphPad Prism we used in this study.

- 191: I would have expected the shoeing procedure to be stressful but this study did not find differences in HR and RR when comparing pre and during time periods. This would actually indicate that they are both not stressful procedures. This also makes me wonder where the pre time period was measured, is this done in their stable or in the shoeing box? If the pre measurement is taken in the ‘calm box’ this might make the horses anticipate to the shoeing before the actual treatment starts and might cause psychological stress. This may also explain why you do not find differences between pre and during periods. If the post period measurements are done within their own stable this might also explain the increase in HRV. I also wonder how controlled the post time periods are. Could there be feeding procedures taking place which might influence HRV parameters for example?

Response to the reviewer

As stated above, the term comfort (more comfort) is used instead of stress (lower stress) as horses experience comfort after two shoeing protocols. It is more suitable to use more comfort rather than lower stress in hot-shoed horses that showed higher HRV during 120 minutes after shoeing. In this study, all horses were taken RR interval data while waiting at the “shoeing box” for 30 minutes before their shoeing. After finishing the shoeing protocol, the horses were returned to their stable. Only water and hay were provided as usual in their stable. Pellets and physical activity were paused for 120 minutes after shoeing.

- Results section in general: is there added value in providing all these parameters in both time, frequency and non-linear variables? Why for example not only provide the SD1/SD2 ratio for the non-linear variables.

Response to the reviewer

This study aimed to investigate the modulation of HRV in horses following shoeing protocols and compared the HRV modification between horses receiving hot or cold shoeing protocols. All parameters were computed from RR interval data by the Kubios Scientific program. So, no additional value or data other than the Kubios program to generate HRV variables. Regarding the nonlinear variable, the SD1/SD2 ratio reflects the sympathovagal balance. Unchanged in this ratio means that the modulation in SD1 and SD2 are parallel and show a balance between sympathetic and vagal action during the measurement. However, the expression of separate SD1 and SD2 values is still crucial in this study as we could find the interaction effect of shoeing protocol and time on changes in SD1 and SD2, meaning that the different shoeing protocols exert a distinct impact on changes in HRV variables. This notion should refer to the importance of reporting the separate LF and HF power spectrum rather than only the LF/HF ratio.

- 285: the author explains clearly why only HR measurements are not enough, however no results are discussed against literature references. Therefore I wonder if this Alinea should not be placed to the introduction section?

Response to the reviewer

We do apologize for not having the discussion regarding the HR measurement result. The discussion regarding the present study’s HR measurement has been placed on page 15, lines 295-298.

- 296: see above, the same accounts for this Alinea, the explanation why you used a HRM could also be part of the introduction or material& method section.

Response to the reviewer

We’d like to leave the explanation of why we used the HRM in this discussion part. That’s because we’d like to explain the intention to use the latest version of the Polar HR monitoring setting (polar belt, H10 sensor, and Vantage 2 polar sports watch) along with the latest version of the Kubios program that provides autonomic correction (noise and artifact correction that are provided only in the premium program). The explanation in this section is expected to convince the reader of the reliable result of this study.

- 310: ‘more precise’ compared to what?

Response to the reviewer

We do apologize for giving unclear information. We’ve modified the text as “the HRV variables in the present study could have reflected precise ANS regulation in response to the shoeing of these horses” on page 16, lines 313-314.

- 326: The author suggests that horses experiences stress relief in the hooves following hot shoeing, however it could also be that during the pre and during time period the horses were anticipating and stressed about the shoeing process, which decreased during the 30 min. time period. Not so much indicating physiological stress relief but reduction in psychological stress. Please substantiate this statement with literature and also consider other possible explanations.

Response to the reviewer

We do apologize for giving an unclear message. Previously, we stated in lines 325-326 that “these results suggested, in general, that horses experienced stress relief following both shoeing practices,” not hot shoeing practice. Typically, the stress index, deriving from the Kubios Scientific program (premium version), is the square root (to make the index normally distributed) of Baevsky’s stress index used for the determination of the stress level and estimation of the body adaptability standards of measurements and physiological interpretation according to the Kubios user’s guidelines. So, we couldn’t indicate whether it is psychological or physiological stress. As we stated, “stress relief in the hooves” may be exaggerated, so we modified the text from “ stress relief in the hooves to “stress relief” as “These results suggested, in general, that horses experienced stress relief following both shoeing practices” on page 16, lines 329-330

- 338: There are also indications in literature that you should adapt the frequency band thresholds for horses, please substantiate your choice further and discuss this in more depth.

Response to the reviewer

As stated above, Diana Stuck et al. (2015) (https://doi.org/10.1016/j.applanim.2015.02.007), reported that several threshold settings are used for spectral analysis. There are a lot of studies using the frequency threshold for horses. However, Gehrke, E.K., Baldwin, A. and Schiltz, P.M. (2011) conducted research in horses adopting frequency threshold for analyzing spectral power in humans (https://doi.org/10.1016/j.jevs.2010.12.007). In addition, another study from our team has implemented the human frequency threshold for frequency domain analysis (https://doi.org/10.1016/j.jevs.2023.104934). In our opinion, the application of the human frequency threshold is acceptable as long as there is no quantitative comparison with other HRV studies but within the current investigation.

- 348: pleas rephrase this line and replace the word excellent, for example by: “These results indicate a larger contribution of sympathovagal activity…..”

Response to the reviewer

We’ve rephrased the context according to the reviewer's comment on page 17, lines 352-253, accordingly.

- 378-379: how an this increased vagal activity at 30 min. and increased sympathovagal activity at 60-120 min. be explained, did you control for activity/locomotion behaviour for example? It could mean that the horses were less active at 30 min. showing more resting behaviour and more active at 60 min. but is this due to the shoeing method or could there be differences in regime at the several housing locations for example feeding practices?

Response to the reviewer

In fact, horses returned to their stable within less than 5 minutes after finishing shoeing. So, it is assumed that individual horses' housing conditions, activity, and other factors were similar throughout the 120-minute period after shoeing. No feed was given during the 120 period in the stall. From the result, it is expected that horses may derive the most comfort 30 minutes after hot shoeing and show more comfort 60-120 minutes after shoeing compared to before shoeing.

- 379-382: during what time period did you observe no change in SD2/SD1 and HF/LF ratio? “….supported the observance of the integral generation of…..” this a bit vagely formulated. The paragraph is well explained in general and shows nice depth in the discussion. However this last sentence needs some work.
- Response to the reviewer

Since the SD2/SD1 and LF/HF ratios indicate sympathovagal balance in response to the stimuli. An increase in those ratios reflects a shift toward sympathetic activity. However, the increase in this ratio may result from an increase in the proportion of SD2 and LF or a decrease in SD1 and HF. An increase in multiple HRV variables that are influenced by both sympathetic and vagal components was observed in the present study. The unchanged SD2/SD1 and LF/HF ratios throughout the study may reflect that the increase in HRV in the current analysis was equally contributed by both autonomic nerve components, not mainly parasympathetic impulses. We’ve modified the text to give more precise information on page 18-19, lines 383-386.

- 386-388: I would have expected differences between pre and during shoeing methods, how can you explain that you did not find this? Did you “assume” or do you mean “hypothesize”, and would you expect the different shoeing methods to show no differences?

Response to the reviewer

This study investigates the change in HRV (as objective evaluation) in response to different conventional shoeing protocols. In practice, horses receive shoeing protocol with their familiar method (hot or cold shoeing) and farrier. Moreover, traditional shoeing on healthy horses by professional farriers isn’t considered a stressful condition unless done on lame horses or shoeing in horses with unhealthy or injured hooves. So, we are not surprised that there is no difference in autonomic responses between pre- and during shoeing as no psychological stress is expected. However, we are thrilled to witness the increased HRV in horses after hot shoeing and show higher HRV value than horses after cold shoeing. The searing of the hoof wall before shoeing caused a reduced uneven surface of the hoof wall and improved juxtaposition, causing appropriate contact surface between the hoof wall and metal shoes in horses with hot shoes. It makes sense to get horses more comfortable after hot shoeing.

392: remove the word excellent

Response to the reviewer

We’ve removed it regarding the reviewer's comment accordingly.

- 393-394: previously you stated that you expected no differences but this sentence contradicts it since here you state that you expect horses to show lower stress levels. The phrase “be lower stress” is not correctly formulated, please replace it with: show lower stress levels.

Response to the reviewer

We’ve modified the text by using “more comfort” instead of “lower stress level” on page 19, line 397 as horses get comfort after shoeing. A higher HRV in hot-shoed horses should be considered more comfortable than lower stress.

- 397: this study does not investigate the reliability of the measurement, perhaps replace it with the usefulness/applicability? And what do you intend to measure: psychological stress or physiological stress? Since later in the paragraph you mention that shoeing can affect the horse’s gait so perhaps the time effect is not so much due to stress but to locomotion?

Response to the reviewer

We’ve modified the text according to the reviewer’s comment on page 19, line 401. Genuinely, the physiological stress response was investigated in this study. However, we avoid using stress in this manuscript as it conveys an unclear message to the reader. We’ve used autonomic response instead of stress response, which may be more suitable and get readers no confusion on page 19, line 401. Regarding the last question, we measure the HRV modulation during shoeing and resting after shoeing. In this connection, we found an interaction effect between shoeing protocol and time on change in HRV. So, time still affects HRV modulation, reflecting stress responses to the stimuli in the horse. Since we’ve reported the HRV modulation at rest in response to shoeing protocols, further investigation of HRV in horses during exercise or activity after shoeing is needed, as we proposed in lines 414-419.

- 402: ‘…..continued study ….is required in athletic horses’, you only studied HRV variables so in my opinion the author cannot make inferences from this on locomotion and exercise as a parameter. Although it might have been a good idea to also take locomotion into account for this study as a parameter.

Response to the reviewer

As we investigate the effect of the shoeing protocol on HRV modulation at rest. We proposed further study on the impact of the shoeing protocol on HRV modulation during exercise, as stated in lines 402-407, which may help investigate the effectiveness of the shoeing protocol on exercise performance using the HRV parameters.

- 404: limitations of the study do not discuss the fact that you might have a bias in your results as the hot shoeing procedures are applied to jumping horses and horses from riding schools, whereas cold shoeing was applied to polo horses, this might affect your results due to differences in training, body condition, character etc.

Response to the reviewer

As we experimented with the effect of shoeing protocol on HRV modulation at rest, not the exercise or other interventions, the difference in sports disciplines, body condition, training, etc., wouldn’t affect the result but the effect of the shoeing protocol. This notion was supported by no difference in HRV variables between horses receiving hot and cold shoeing at pre-shoeing. So, the variation in factors the reviewer mentioned is expected to have no impact on the HRV modulation in this study.

- 405: stress does not lead to errors in HRV but psychological stress affects your HRV parameters just as much as physiological stress. Did you record which horses were treated with an unfamiliar type of horseshoeing and check whether this factor had an effect?

Response to the reviewer

We’ve modified the text regarding “stress does not lead to errors in HRV” to convey the appropriate message on page 19, lines 408-409. In this study, we haven’t checked whether shoeing with unfamiliar types because it is out of our objective. it may be predicted that horses accustomed to hot shoeing may not show signs of anxiety or fear when doing cold shoeing. However, the horses used to fitting with cold shoes are expected to show fear or anxiety when burning shoe sears on the hoof wall; moreover, the grey smoke from the searing may frighten them, causing an increase in either psychological or physiological stress levels. For this reason, we haven’t conducted the experiment in crossover design in this study.

- 407: the length of the time frame might also affect some of your HRV parameters, did you control for this and were all time periods of the same duration?

Response to the reviewer

Yes, the HRV in all horses was determined 30 min before shoeing, during shoeing (took 45-60 min) and at 30 min intervals after shoeing for 120 min as shown in tables 1-3. So, the time periods were controlled and taking the HRV variables (except during shoeing) at the same duration.

- 413: ‘although the horse applying….”sentence is not correctly formulated and therefore unclear

Response to the reviewer

We do apologize for giving unclear information. We’ve modified the text to provide more explicit information on page 20, lines 417-421.

- 416: I wonder what the definition of stress is in this study, do you mean psychological stress or physiological stress?

Response to the reviewer

We do apologize for giving the reviewer unclear information. We avoid using stress in this manuscript and context has been modified on page 20, lines 417-421.
