# Supplementary material for: Heart rate and heart rate variability in horses undergoing hot and cold shoeing
Source: PLoS One. 2024 Jun 6;19(6):e0305031. doi: 10.1371/journal.pone.0305031 (PMC11156413; doi:10.1371/journal.pone.0305031)
Supplement: Attachment — Submitted filename: Feedback 2 author.docx [file pone.0305031.s005.docx]

- In general the introduction is greatly improved and clearly explained.
- I appreciate the attempt to clarify the purpose of the study. However, the issue is not resolved by changing the term ‘stress’ into ‘comfort’ in the conclusion. The point here is that in this study you are actually aiming to measure a stress response (line 46-50) and not (just) physiological activity. However, you cannot distinguish between physiological stress and psychological stress in this study, as you have not controlled for physical activity and did not record behavioural parameters. This makes interpretation of the results and differences between time periods a bit more difficult. Both psychological and physiological stress affect HRV and may be reflected in physiological parameters of stress. Therefore the authors conclusion that hot shoed horses experience less discomfort/stress should be a bit more carefully formulated (less straightforward). Perhaps hot shoed horses spend more time standing still after the shoeing process which leads to decreased HRV parameters? This may still be an indication of discomfort, so I do not disagree with the conclusion, but it could be formulated a bit more carefully. Even more so since the differences between the methods could also be due to differences in breed/character, body condition, differences in stable size etc…..due to the setup of the study. Suggestion line 416-: ‘ANS activity responds differently in horses receiving different shoeing methods. Higher HRV 30-120 minutes after hot shoeing suggests that hot-shoed horses experience less stress compared to cold-hoed horses after shoeing. More research is needed to investigate how different shoeing techniques affect stress levels in horses.’
- In this study the shoeing process itself does not lead to increased HRV parameters compared to the pre-period. This might indicate that horses do not experience psychological stress from either procedure. This is in contrast to what I would expect, but could be due to the fact that horses are accustomed to the process already and because you selected them for easy shoeing (creating a bias). Therefore mention this in your M&M section because it puts your results into perspective. If you would change the inclusion criteria you might find different results as some horses would indeed experience shoeing as a stressor. However, it also depends on how the pre measurement is taken, if this is already done within a restraint box (or a separate place where the shoeing takes place) the horses might already anticipate to the procedure and experience psychological stress. This needs to be addressed in the discussion. It would have been preferable if the pre measurement had been done in the stable so the conditions of pre and post would be comparable.
- Remark 85: mention in your results the selection process of the horses and that they are accustomed to wearing a girth.
- Remark 93: I assume that the farriers work in certain stables and also perform either hot or cold shoeing so you did not control for this? This also means you cannot distinguish the effect of the shoeing procedures from the farrier and/or location effects so this makes it difficult to interpret the results. Please also explain this in detail in your M&M.
- Remark 105+110+111 mention this in the M&M section
- Remark 191: See also previous remarks. The horse might be simply more comfortable in the stable compared to the ‘calm box’ and this does not necessarily have to be caused by the shoeing process. If you want to talk about stress/comfort during the post period you should be able to compare this to a pre period in which the horse is within the same conditions (so its own stable). I really think that the term ‘calm box’ is misleading, if this is the station where the horses are normally shoed than this location might actually be associated with stress. This might explain the increase in HRV in the post period compared to the pre period but not the fact that this difference was not found in the cold-shoeing method. However, the differences between the two shoeing methods during the post period may also be caused by differences in location (for example bedding) due to the setup of the study. This actually presents two methodological problems that needs to be addressed in the discussion at least. Actually location and farrier should have been controlled for or included as a factor in your model. This cannot be changed anymore but it should be considered when formulating the conclusions and mentioned in the discussion.
- Remark 404: this bias cannot be disregarded that easily as the type of horse (selection for sport purposes) also determines their character and stress responsiveness and/or recovery after a stressful event such as shoeing. Again the changes between hot shoed and cold shoed horses might not only be due to shoeing differences but also to differences in stable size or type of horses between locations etc.

Remarks on the new version with corresponding line numbers;

- Line 276: ‘and time on multiple HRV variables’
- Line 287-299: is a well written explanation why you chose to look at HRV and not only HR, but this is not a discussion that compares your results to existing literature. It would better fit within the introduction or should be relating to your findings and findings in literature.
- Line 300-310 explains why you would use a HRM monitor, this again is not a discussion that reflects on findings but should be placed into the introduction and/or M&M section or should be relating to your findings and findings in literature. Whereas lines 310-313 are a result.
- Line 330: ‘stress relief’ should be replaced by reduced stress or stress reduction
- Line 397: Remove ‘Accordingly……hot shoes.’ The previous line is more objective and already discusses the increased sympathovagal activity.
