# Supplementary material for: Heart rate and heart rate variability in horses undergoing hot and cold shoeing
Source: PLoS One. 2024 Jun 6;19(6):e0305031. doi: 10.1371/journal.pone.0305031 (PMC11156413; doi:10.1371/journal.pone.0305031)
Supplement: Attachment — Submitted filename: Response to reviewers.docx [file pone.0305031.s006.docx]

**Heart rate and heart rate variability in horses receiving hot shoeing and**

**cold shoeing**

**Dear Editor and reviewers,**

I am grateful for offering to revise this manuscript. We’ve addressed all points raised by

the reviewers and the modifications in the text were highlighted in green.

**Response to the reviewer 2#**

- In general, the introduction is greatly improved and clearly explained.

Response to the reviewer

We would like to thank the reviewer’s kind words.

- I appreciate the attempt to clarify the purpose of the study. However, the issue is not resolved by changing the term ‘stress’ into ‘comfort’ in the conclusion. The point here is that in this study you are actually aiming to measure a stress response (line 46-50) and not (just) physiological activity. However, you cannot distinguish between physiological stress and psychological stress in this study, as you have not controlled for physical activity and did not record behavioural parameters. This makes interpretation of the results and differences between time periods a bit more difficult. Both psychological and physiological stress affect HRV and may be reflected in physiological parameters of stress. Therefore, the authors conclusion that hot shoed horses experience less discomfort/stress should be a bit more carefully formulated (less straightforward). Perhaps hot shoed horses spend more time standing still after the shoeing process which leads to decreased HRV parameters? This may still be an indication of discomfort, so I do not disagree with the conclusion, but it could be formulated a bit more carefully. Even more so since the differences between the methods could also be due to differences in breed/character, body condition, differences in stable size etc…..due to the setup of the study. Suggestion line 416-: ‘ANS activity responds differently in horses receiving different shoeing methods. Higher HRV 30-120 minutes after hot shoeing suggests that hot-shoed horses experience less stress compared to cold-hoed horses after shoeing. More research is needed to investigate how different shoeing techniques affect stress levels in horses.’

Response to the reviewer

We would like to thank the reviewer's comment and explain this study briefly. HRV is generally adopted to indicate autonomic regulation in which a reduced HRV reflect increased stress in such circumstances as pain, exercise, transportation, etc. Regarding a subjective evaluation and anecdotal evidence, the practitioner and farrier think that shoeing with hot shoes is better than cold shoeing as it improves juxtaposition between the hoof surface and metal shoe as well as burning the hoof solar surface to close the hoof tubule, leading to preventing the invasion of microorganism to the hoof wall via these tubules. However, there is no objective evidence to prove this belief. This led us to apply the HRV to indicate the autonomic regulation to objectively investigate whether or not horses undergoing hot shoeing derived more comfort than cold shoeing. In this study, we try not to use the stress response in the whole text but in the introduction to avoid misconducting since shoeing experienced horses isn’t supposed to be a stressor, as we discussed on **page 16, lines 321-325**.

Regarding the reviewer's comment about distinguishing between physiological stress and psychological stress, we didn’t aim to differentiate between both types of stress but rather the overall autonomic response following different shoeing types. However, we designed the study to minimise psychological stress by 1) experimenting on horses that are accustomed to fitting with each type of farrier practice and 2) shoeing horses by familiar farriers and places. **By doing this, the changes in HRV variables in this study were expected to result mainly from different physiological stimuli following hot and cold shoeing**. We’ve added this document in the material and methods section on **page 5, lines 104-106**. As measured before shoeing, **HRV variables didn’t differ between HSH and CSH groups, implying that horses in both groups showed similar autonomic responses** regardless of the type of physical activity, sex, breed, etc., before shoeing (as shown in tables 1-3). We’ve added this information in the discussion part on **pages 14-15, lines 283-287**. Of note, the behavioral parameter is not practical in this study because conventional shoeing in healthy horses carried out by familiar farriers may not trigger behavioral changes during the shoeing period, and it might not be suitable for assessing the autonomic response to shoeing methods in horses (**as stated on page 3, lines 45-49**). That’s why we haven’t implemented the behavioral measurement in this study.

A major difference between both shoeing types is that heating the metal shoe, searing the hoof solar surface and adjusting the shoes to fit the solar surface were performed in hot shoeing. On the other hand, the metal shoes were directly adjusted to fit the solar surface without heating in cold shoeing. In fact, heating shoes aids in metal shoe adjustment in hot shoeing and takes less time than adjusting non-heating shoes in cold shoeing. So, the time taken for horseshoeing was somewhat similar in both groups, and horses fitting with hot shoes didn’t take longer to stand still than horses fitting with cold shoes. Each horse was walked to their stable after completing the shoeing protocol. This is due to the fact that overall HRV in this study was either constant throughout the study or increased during and after shoeing. A decrease in “some HRV variables, such as stress index and SNS index” indicated a positive impact of such shoeing.

Considering the HRV modulation during shoeing, there were almost no changes in HRV variables (except stress index, which was reduced during shoeing) during shoeing but increased at around 90-120 minutes after shoeing in the CSH group. **These results suggested that CSH horses were not under stress conditions during and after shoeing**. In contrast, various HRV variables increased in HSH horses during shoeing, extending to 120 minutes after shoeing. Moreover, multiple HRV variables were higher in HSH than CSH horses at given time points after shoeing. An increase in HRV reflected more relaxation or comfort in HSH horses. That’s why we avoid using the terms less stress, reduced stress, or minimal stress in horses receiving shoeing but apply the term “more comfort” following an increase in HRV in horses receiving hot shoeing. This result implies that **HSH horses not only showed no stress conditions during shoeing but also derived more comfort after shoeing and demonstrated more comfort than CSH horses after shoeing**.

- In this study the shoeing process itself does not lead to increased HRV parameters compared to the pre-period. This might indicate that horses do not experience psychological stress from either procedure. This is in contrast to what I would expect, but could be due to the fact that horses are accustomed to the process already and because you selected them for easy shoeing (creating a bias). Therefore mention this in your M&M section because it puts your results into perspective. If you would change the inclusion criteria you might find different results as some horses would indeed experience shoeing as a stressor. However, it also depends on how the pre measurement is taken, if this is already done within a restraint box (or a separate place where the shoeing takes place) the horses might already anticipate to the procedure and experience psychological stress. This needs to be addressed in the discussion. It would have been preferable if the pre measurement had been done in the stable so the conditions of pre and post would be comparable.

Response to the reviewer

We would like to thank the reviewer's comment and explain a little bit about the study protocol. In this study, the psychological stress was expected to be eliminated by the study design, as stated on **page 5, lines 104-106**. we confirm that we didn’t aim to create bias but designed the experiment to “minimize psychological stress” by 1) experimenting on horses that are accustomed to fitting with each type of farrier practice and 2) shoeing horses by familiar farriers and places. If we’ve selected horses who aren’t accustomed to the farrier practice (new horses or horses that haven’t been shod before, for example) or have designed the experiment as the cross-over design (one horse that is only familiar with either hot or cold shoeing receives both shoeing protocols), we could have created “the predictable stressor”, leading to decreased HRV and increased stress due to fear and anxiety in those horses and the results cannot reflect the effect of shoeing but the fear or anxiety on autonomic modulation. However, to give more precise information, we’ve added the document on why we selected the experience horses for shoeing in this study in the material and method part on **page 5, lines 95-96 and 104-106**.

In the present study, the pre-shoeing measurement was done in designated stables within the farrier place for 30 minutes before leading them to be shod in the calm station in front of the designated stables (figure 1) or next to the designated stable (figure 2). The waiting stable is similar to the horse stable in terms of stable type, design, size, and straw bedding. Horses were allowed to have hay and drink water as if they were housed in their stable before shoeing. For this reason, HRV values at given time points and pre-shoeing values could be comparable. This message has been added to provide more precise information on **page 6, lines 114-117**.

Supposing that shoeing for experienced horses is a non-stress condition. Still, it should be “a reliever “, as the shoeing method is conducted to correct hoof discomfort during the late period of 4-6 weeks of scheduled shoeing. Since the hoof wall grows over time, the increased weight-bearing length following toe growth and a decreased heel angle may cause hoof angle displacement. Moreover, metal shoes do not change the size, and the maximum expansion of the hoof wall is limited. These events cause gradual discomfort in the shod feet of horses, as we discussed **on page 16, lines 321-324**.

Not surprisingly, the shoeing protocol is supposed to relieve discomfort, as indicated by reduced stress index and sympathetic nervous system index in horses during shoeing with both protocols. More importantly, the differences in HRV modulation between shoeing types were observed in horses after shoeing, reflecting that autonomic response differed between horses receiving distinct farrier practices. If shoeing had been the stressor in experienced horses, the HRV could have been reduced (not increased or remained constant), indicating increased stress during shoeing.

- Remark 85: mention in your results the selection process of the horses and that they are accustomed to wearing a girth.

Response to the reviewer

We would like to thank the reviewer's comment. We’ve added the information regarding the criteria for horse selection in this study on **page 5, lines 95-99 and lines 104-106**. As per our routine protocol, the HRM device was equipped on horses for at least 10 minutes to get them accustomed to the setting before the recording started. We’ve added this sentence on **page 6, lines 114-117**.

- Remark 93: I assume that the farriers work in certain stables and also perform either hot or cold shoeing so you did not control for this? This also means you cannot distinguish the effect of the shoeing procedures from the farrier and/or location effects so this makes it difficult to interpret the results. Please also explain this in detail in your M&M.

Response to the reviewer

We would like to thank the reviewer's comment. Suppose we organised the controlled shoeing protocol by assigning only one farrier to shoe all horses or arranged them to shoe horses in equal numbers and shoeing protocols (regardless of familiarity). In that case, many horses may have experienced anxiety, fear or uncertainty, causing distortion in HRV modulation due to anxiety and fear rather than the shoeing protocol. Similarly, staying all experiment horses in the same designated places before, during and after shoeing may have caused anxiety and fear, predisposing them to stress from unfamiliar locations and distorting the HRV modifications.

As stated earlier in this response to the reviewer and within the text on **page 5, lines 95-96 and 104-106**, each horse received the shoeing protocol by familiar farrier, method and place. In fact, both shoeing protocols were conducted virtually all the same unless the seared solar surface was before adjustment and nailing in hot shoeing and nailing directly after adjustment in cold shoeing. Moreover, shoeing, in reality, causes no pain as all cutting, floating and nailing were done on the insensitive hoof wall by professional farriers, as it is evident that no HRV variables were reduced during shoeing. In addition, the condition of waiting stable for shoeing was similar to their stable (stable type, size and bedding type). So, the arrangement in this study aimed to avoid or minimise the “psychological factor” from shoeing by unfamiliar farriers and locations during the shoeing. Thus, changes in HRV variables were expected to result from horses' physiology (discomfort, remaining neutral or more comfort) during and after shoeing in horses. We’ve also discussed this issue on **pages 14-15, lines 283-290**.

- Remark 105+110+111 mention this in the M&M section

Response to the reviewer

We do apologize that we didn’t catch this reviewer's comment. As lines 105, 110 and 111 are in the material and methods section.

- Remark 191: See also previous remarks. The horse might be simply more comfortable in the stable compared to the ‘calm box’ and this does not necessarily have to be caused by the shoeing process. If you want to talk about stress/comfort during the post period you should be able to compare this to a pre period in which the horse is within the same conditions (so its own stable). I really think that the term ‘calm box’ is misleading, if this is the station where the horses are normally shoed than this location might actually be associated with stress. This might explain the increase in HRV in the post period compared to the pre period but not the fact that this difference was not found in the cold-shoeing method. However, the differences between the two shoeing methods during the post period may also be caused by differences in location (for example bedding) due to the setup of the study. This actually presents two methodological problems that needs to be addressed in the discussion at least. Actually location and farrier should have been controlled for or included as a factor in your model. This cannot be changed anymore but it should be considered when formulating the conclusions and mentioned in the discussion.

Response to the reviewer

We would like to thank the reviewer's comment. In this study, the HRV metrics were measured and computed 30 minutes pre-shoeing at designated stables where the conditions were similar to their stable, as stated earlier in this reviewer’s responses. Horses have shod in calm stations in front of the stable (as shown in Figure 1) or next to the designated stable (as shown in figure 2). In fact, all horses were usually housed in a similar standard box size (3x5 m^2^) with straw bedding. The designated stable for horses waiting for shoeing is similar to the housing stable, including bedding. As the reviewer gave us a comment that “The horse might be simply more comfortable in the stable compared to the ‘calm box’, and this does not necessarily have to be caused by the shoeing process”, it would have been true if we found an increase in HRV after shoeing (when they were in their own stables) “in horse receiving both shoeing types.” In fact, the increased HRV was found mainly in hot-shoed horses even though all conditions of the housing stable for horses in both shoeing types were somewhat similar, including **straw bedding type**. This result could reflect different autonomic responses following physiological factors in horses with different shoeing protocols. We’ve added the details in the material and method that the study was designed to avoid or minimise the psychological stress on pages **5, lines 94-95 and 104-106** and have discussed more on this issue on **pages 14-15, lines 283-290**.

- Remark 404: this bias cannot be disregarded that easily as the type of horse (selection for sport purposes) also determines their character and stress responsiveness and/or recovery after a stressful event such as shoeing. Again the changes between hot shoed and cold shoed horses might not only be due to shoeing differences but also to differences in stable size or type of horses between locations etc.

Response to the reviewer

We would like to thank the reviewer's comment. As stated in the discussion part (**on page 15, lines 288-289**), the HRV variables that were computed before shoeing protocols didn’t differ between HSH and CSH groups (Tables 1-3). These results implied that horses in both groups showed similar autonomic responses before the commencement of the study regardless of sex, activity type or location. As we’ve stated on **page 5, lines 89-90 and page 6, lines 114-116**, all conditions of the housing stable for horses in both shoeing types were somewhat similar, including straw bedding type. Moreover, no typical activities were involved in this shoeing study except waiting in the designated stable, shoeing, and returning to the housing stable. In addition, horses were shod with their familiar farriers, methods and places. This arrangement was expected to minimise the effect of such factors, causing distortion in HRV variables, and the HRV variables were modulated according to the physiological factor following different shoeing methods.

- Remarks on the new version with corresponding line numbers;

Response to the reviewer

We would like to thank the reviewer's comment. We’ve rechecked and added the line number already.

- Line 276: ‘and time on multiple HRV variables’

Response to the reviewer

We would like to thank the reviewer's comment. We’ve corrected it by using “affected” on **page 14, line 271** according to the proofreading service correction before 2^nd^ revised submission.

- Line 287-299: is a well written explanation why you chose to look at HRV and not only HR, but this is not a discussion that compares your results to existing literature. It would better fit within the introduction or should be relating to your findings and findings in literature.

Response to the reviewer

We would like to thank the reviewer's comment. In this context, we want to let the reader realise that HR was not appropriate to measure autonomic regulation as the interplay between PNS and SNS components influences it. In this study, as mentioned in the discussion part on **page 15, lines 299-302,** the HR didn’t differ between pre- and during-shoeing, but the difference was found among during-shoeing and post-shoeing time points in both groups. Also, the difference between groups at each time point was not observed. So, the HRV variable for indicating PNS and SNS activity would be important to quantify the particular autonomic responses in horses receiving different shoeing types.

- Line 300-310 explains why you would use a HRM monitor, this again is not a discussion that reflects on findings but should be placed into the introduction and/or M&M section or should be relating to your findings and findings in literature. Whereas lines 310-313 are a result.

Response to the reviewer

We would like to thank the reviewer's comment. It would be true that we want to let the reader know why we use HRM devices even though the use of electrocardiogram (ECG) monitoring was considered the gold standard for HRV analysis. In this context, we want to convince the reader that the HRV analysis in this study is reliable and produces acceptable HRV variables by using this HRM device. As errors and artefacts were inevitably observed when using the HRM device, we want to address the use of the reliable HRV computed program to correct the errors and artefacts more effectively. In addition to the device and program, we want to let the readers know that horses seem to be in no discomfort and that the device installation does not interfere with the shoeing practice following subjective evaluation by the vet who witnesses and supervises the shoeing protocol as stated on **pages 15, lines 314-317**.

- Line 330: ‘stress relief’ should be replaced by reduced stress or stress reduction

Response to the reviewer

We would like to thank the reviewer's comment. This words were proposed by the English proofreading service; however, we’ve modified it according to English proofreading service on **page 16, line 332**.

- Line 397: Remove ‘Accordingly……hot shoes.’ The previous line is more objective and already discusses the increased sympathovagal activity.

Response to the reviewer

We would like to thank the reviewer's comment. We’ve already removed it according to the reviewer comment.

**Response to the reviewer #3:**

Introduction

I do understand the aim of the study, however I advise authors to re-work the Introduction Section. Authors do explain why shoeing is important. However, both shoeing methods could be explained further to readers. Then, the need for this study on stress should be more detailled: why do authors expect stress when shoeing? I believe the whole paragraph on HRV methods (lines 62-90) can be shortened to one or 2 sentences. There is no need to introduce the different HRV methods in such an extend. Furthermore, looking at the explanations of authors on HRV methods, I wonder if all HRV methods were needed for this study. Also, not all HRV parameters have been validated in horses.

Response to the reviewer

We would like to thank the reviewer's comment. In fact, routine shoeing by professional farriers is expected to cause no stress; rather, it offers horses comfort after shoeing because it corrects the hoof imbalance, as we added to the discussion on **page 16, lines 321-325.** This study aims to provide objective evidence to evaluate whether or not hot shoeing is better than cold shoeing based on previous subjective evaluation, as described in the introduction on **page 3, lines 40-43.** There is a lot of literature adopting the HRV, which is modulated following radical challenges, to quantify the autonomic response in horses in several aspects. So, we employed this advantage of using HRV modulation to indicate the autonomic response in horses receiving different types of shoeing protocols. More importantly, as fundamental shoeing is expected to cause no stress, as stated above, we use the term “autonomic response or ANS responses” rather than “stress response” in this main text to avoid misleading the result of this study’s experiment.

According to the explanation of different HRV methods, we retained it in the introduction part as we are concerned that readers from outside the HRV field (in particular, farrier and practitioners in the hoof and shoeing field) may not understand the use of HRV variables to indicate ANS responses in this study. However, we still modified it, and the section was shortened from 415 words to 220 words on **page 4, lines 61-76**.

Concerning the HRV parameter, there haven’t been reports on the HRV modulation to indicate the autonomic response in horses receiving the different types of shoeing.	In this regard, we aimed to demonstrate all HRV analysis methods to convince the readers that our results were reliable in indicating HRV modulation in this study. For example, we measured the time domain HRV variables to evaluate whether or not they changed according to the vagal activity or both components (sympathetic and vagal activity) (<https://doi.org/10.1016/j.applanim.2015.02.007> and <https://doi.org/10.1016/j.physbeh.2007.01.007> ). Later, we applied frequency domain analysis to estimate the sympatho-vagal balance, which cannot be properly assessed by the sole time domain analysis (<https://doi.org/10.1016/j.applanim.2015.02.007>). Finally, we adopted nonlinear analysis to measure short-term HRV under the influence of vagal activity (SD1) and long-term HRV under the influence of both vagal and sympathetic impulses (SD2). Based on the results by using 3 HRV analysis methods, we can summarise that increased HRV mainly result from the vagal component at 0-30 minutes after shoeing (increased RMSSD and SD1) followed by both vagal and sympathetic components at 30-120 minutes (increased in SDNN, LF, TINN, HRV triangular index and SD2). Although many readers think that some variables may not be validated in horses, all variables in this study have been reported to indicate HRV in horses in other studies. (<https://doi.org/10.3390/ani11030693>, <https://doi.org/10.1016/j.tvjl.2020.105590>, <https://doi.org/10.12688/f1000research.17997.1>)

Material and methods

- Line 111-113: do authors mean complications after shoeing during experimental protocol? In this case, it cannot be an inclusion criteria as horses are already in the protocol. If authors mean previous shoeing, please mention. If horses were excluded after inclusion due to lameness after study shoeing, please indicate how many horses were excluded and for which reason (even if n = 0).

Response to the reviewer

We would like to thank the reviewer's comment. We indeed meant the complications after shoeing. We’ve taken off the sentence “, and (4) there was no lameness during straight-line trotting on soft ground before and after farrier practice” from the inclusion criteria and the main text was modified on **page 5, lines 99-100**.

- Experimental design: please indicate where the horses were kept before and after shoeing, as HRV measurements depends greatly on the way horses are kept (see many studies in equine literature). The standardization of the data collection is one major effect on HRV measurements.

Response to the reviewer

We would like to thank the reviewer's comment. More detail in experimental design has been added in the material and method on **page 5, lines 104-106**.

- Experimental design: how can authors exclude an effect of shoeing depending on the individuals? There is no consensus about HRV values in horses, and studies show largely different values depending on horses. Why did authors choice for this study design and not for example a crossover design?

Response to the reviewer

We would like to thank the reviewer's comment. In this study, we aimed to eliminate or minimise the psychological stress by 1) experimenting on horses that are accustomed to fitting with each type of farrier practice and 2) shoeing horses by familiar farriers and places as stated on **page 5, lines 104-106**. If we’ve selected horses who aren’t accustomed to the farrier practice (new horses or horses that haven’t been shod before, for example) or have designed the experiment as the “cross-over design” (one horse that is only familiar with either hot or cold shoeing receives both shoeing protocols), we could have created “the predictable stressor”, leading to decreased HRV and increased stress due to fear and anxiety in those horses and the results cannot reflect the effect of shoeing but the fear or anxiety on autonomic modulation.

In the present study, the pre-shoeing measurement was done in designated stables within the farrier place for 30 minutes before leading them to be shod in the calm station in front of the designated stables (figure 1) or next to the designated stable (figure 2). The waiting stable is similar to the horse stable in terms of stable type, design, size, and straw bedding. Horses were allowed to have hay and drink water as if they were housed in their stable before shoeing. For this reason, HRV values at given time points and pre-shoeing values could be comparable. This message has been added to provide more precise information on **page 6, lines 115-117**.

Supposing that shoeing for experienced horses is a non-stress condition. Still, it should be “a reliever “, as the shoeing method is conducted to correct hoof discomfort during the late period of 4-6 weeks of scheduled shoeing. Since the hoof wall grows over time, the increased weight-bearing length following toe growth and a decreased heel angle may cause hoof angle displacement. Moreover, metal shoes do not change the size, and the maximum expansion of the hoof wall is limited. These events cause gradual discomfort in the shod feet of horses, as we discussed **on page 16, lines 321-326**.

Before shoeing, there was no difference in HRV variables between CSH and HSH groups (Table 1-3), supposing that all horses were under similar autonomic regulation. In this study, horses followed similar sequences (waiting in the designated stable before shoeing, shoeing in the calm station, and returning to their own stables). The difference in protocol between the groups is shoeing methods (hot or cold shoeing). Unlike doing research in laboratory animals such as mice, rat etc., large variations in individuals are inevitable in field experiments as age, sex, and physical activity cannot be strictly controlled. This could cause an outlier or non-normal distribution of the data. In this study, we used two-way ANOVA with repeated measures provided by the up-to-date version of GraphPad prism (10.1.0), where the Geissier-Green house correction is automatically recommended if equal variability of differences is not assumed, as shown in the picture below. By doing these, the effect on individuals is supposed to be minimised.

We also agree with the reviewer that there is no consensus about HRV values in horses and the quantitative result of HRV in this study cannot be as a reference value in horses. However, the HRV analysis in this study was done in both horse groups at the similar sequence, time period and HRV analysis premium program at the condition we designed to minimise the psychological stress. the expression of HRV variables were potentially comparable between groups to indicate distinct autonomic responses in this study.

- When authors should use less and the most appropriate HRV measurements, authors could use graphs instead of tables for the results, as they present a timeline in measurements.

Response to the reviewer

We would like to thank the reviewer's comment. At first, we designed to demonstrate the results regarding line graphs; however, there have been a lot of figures and sub-panels within the figures. Moreover, the exact mean ± SD value must be shown to the readers where we reported the significant difference p-value within the result part of the main text. Doing this may mess up the text in the result part and cause the reader difficulty in following the result. So, we wish to report the results in tables rather than graphs in this study.

Discussion

- Please indicate HRV measurements are repeatable in horses, both in rest (https://doi.org/10.1016/j.jevs.2016.07.006) and during activities (https://doi.org/10.1163/17552559-20220044), as it may be important to compare groups.
- I do emphasize the efforts of authors to show an effect of shoeing methods on stress in horses, and the need for more knowledge in the current need for improved welfare. However, I am still dubitative about the conclusions authors make with the acquired data. Differences may be significative but are they still related to the shoeing method and are they relevant? I would personally choice for less HRV variables, but better discussed in the whelm of significance, relevance and interpretation. The confounds and biases should me more elaborated in the discussion.

Response to the reviewer

We would like to thank the reviewer's comment. We’ve added the indication recommended by the reviewer on **page 16, lines 317-318**. We’ve also modified the discussion to point out that routine shoeing is not a stressor but a relief, which is evident by no reduction in HRV during and after shoeing compared to before shoeing values. ANS activity responds differently in horses receiving different shoeing methods. The higher HRV after shoeing in hot-shoe horses could reflect that hot shoeing offers more comfort than cold-shoeing in horses. This conclusion doesn’t mean that cold shoeing is inappropriate for shoeing horses; rather it still derive comfort, but the hot shoeing derives more comfort based on the reason we gave in the discussion part.

I would like to read a reviewed manuscript as I do believe this content is of importance for equine science in the context of equine welfare.

**Response to the reviewer #4:**

The article is interesting and well-written. However, there are the following two main points the authors should cope with to meet publication standards.
1. In the preamble of the statistical analysis section, lines 169-173, specify the levels of the two factors under consideration. In particular, how many time points there are? Remember that the two-way repeated measures ANOVA is an omnibus test statistic and cannot tell you which specific groups within each factor were significantly different from each other.

Response to the reviewer

We would like to thank the reviewer's comment. In this study, we use Two-way ANOVA with repeated measures to test whether or not there was an interaction between shoeing type and time on the modification of HRV variables at six time points (before shoeing, during shoeing, 30, 60, 90, 120 minutes after shoeing). Turkey’s *post hoc* was later implemented to test the difference in HRV variable within the group and between the groups at given time points. A statistically significant interaction in this study means that the effect of shoeing type (difference between hot-shoeing and cold-shoeing protocols) on HRV variables differs among time point measurements. After that, we selected multiple comparison tests provided by the GraphPad program to compare differences in HRV variables within groups and between groups at given time points, as shown in the picture below:

Based on a significant interaction effect found in various HRV variables, we summarise that fitting horses with different shoeing types exerts different autonomic responses. Since higher HRV variables reflected lower stress and better flexibility and adaptability of the body system to cope with physical and physiological challenges, the higher HRV after shoeing reflected that HSH horses derived more comfort than CSH horses during this period.

2. A fundamental part of the statistical analysis involves checking that the data can be analyzed using a two-way repeated measures ANOVA. This involves checking if your data "passes" the assumptions that are required for a two-way repeated measures ANOVA to give you a valid result. The most important assumptions to be checked are: 1) there should be no significant outliers in any combination of the related groups, 2) the distribution of the dependent variable in each combination of the related groups should be approximately normally distributed, and 3) the variances of the differences between all combinations of related groups must be equal. Just remember that if you do not run the statistical tests on these assumptions correctly, the results you get when running a two-way repeated measures ANOVA might not be valid.

Response to the reviewer

We would like to thank the reviewer's comment. Normally, a normality assumption is necessary for significant statistical testing using a two-way ANOVA. However, the ANOVA is considered “robust” to deviations from normality, meaning some violations of this assumption can still be tolerated while the test provides valid results. As the sample size increases, the distribution can be non-normal, and the two-way ANOVA can still provide valid outcomes (<https://www.amstatisticalconsulting.com/two-way-anova/>) and (<https://www.graphpad.com/guides/prism/latest/statistics/stat_checklist_2way>anova_rm.htm). Nevertheless, we still applied an algorithm to check that our data passed the assumption and produced a valid result. In this study, we used the GraphPad Prism version 10.1.0, which provides the Greenhouse–Geisser correction. This algorithm provides the benefit of estimating an epsilon (sphericity) and automatically correcting the data's lack of sphericity (as shown in the picture below) when we performed the two-way ANOVA analysis. This adjustment is expected to yield valid results in repeated measures of two-way ANOVA. please correct me if I am wrong.

In this connection, we’ve modified the text in statistical analysis to deliver more precise information on **page 8, lines 161-162**.
