# Supplementary material for: Heart rate and heart rate variability in horses undergoing hot and cold shoeing
Source: PLoS One. 2024 Jun 6;19(6):e0305031. doi: 10.1371/journal.pone.0305031 (PMC11156413; doi:10.1371/journal.pone.0305031)
Supplement: Attachment — Submitted filename: Feedback 3 author.docx [file pone.0305031.s007.docx]

Line 32 (abstract): The conclusion that horses experience more comfort cannot be drawn. Please only state that differences in HRV were found indicating increased sympathovagal activity in horses that were hot shoed compared to cold shoed horses.

Line 45-49: Behavioural parameters can be used to assess stress. This is possible during shoeing practices, for example through recording facial expressions (Ask et al. 2020, van Loon et al. 2015) but also stress related behaviours (Visser et al. 2009, Young et al. 2012). Recording behaviours may have provided more insight into differences in activity. That for this study you decided not to record this for (practical?) reasons is understandable but does not make behaviour an unsuitable parameter. Please move this section to the discussion and add it as a limitation to the study.

Line 143-144: Please indicate which level of noise detection you used and what levels of beat correction were considered acceptable (were no recondings excluded because the recording was to noisy?).

Line 283-286: Please move these lines to the M&M section. The changes in HRV variable cannot be expected to result mainly from physiological factor of the shoeing process as you did not control for differences in location and/or locomotion in the horses. Confounding factors cannot be excluded, for example horses of the cold shoeing location could be anticipating to feeding during your post shoeing measurement explaining the higher HRV compared to hot shoed horses (your M&M section does not give information on differences in feeding times and daily routines). The lack of differences in autonomic responses before the study commenced only eliminates the possibility of individual differences influencing these results.

Line 331-333: Here you use the term stress and not comfort which is confusing for the reader. Furthermore, the conclusion cannot be drawn from your results as your baseline measurement is not carried out under the right circumstances. There is limited evidence that the shoeing process provides comfort and you do not substantiate the statement that gradual discomfort may arise in shod feet. In addition, you do not consider that the process of shoeing might also be stressful. As stated before horses are measured not in their own stable during baseline conditions, placing them in a different stable might already be a stressor, even more so since they may be anticipating to be shod. The only measurement that is done in their own stable is the post shoeing measurement. This may explain the increase in vagal tone as well and does not necessarily have to be contributed to the comfort of the new shoes. This should be discussed and mentioned as a limitation.

Line 397-409: The subjective reporting of more benefits of hot-shoeing compared to cold shoeing is not really something that you can use as a starting point of this alinea. The first sentence can be used as an explanation but not as a fact, so it is better to begin with your own findings (line 399-400) and then state that this is in line with subjective evidence that seems to indicate that hot shoeing is more beneficial that cold shoeing. In this way you also avoid drawing hard conclusions on this subject which cannot be validated by your own research. As stated before confounding factors may contribute to the differences between hot and cold shoed horses as well, so the statement that this study provides objective evidence for greater benefits of hot shoeing should be done more carefully. Especially since you also state in lines 404-407 adverse effects and risks of lameness through shoeing practices you seem to contradict the previous assumption that (hot) shoeing should lead to more comfort.

Line 410: Start this alinea with the statement that a limitation of the study was that a cross-over design was not used and then explain why this is not possible.

Line 420: The line ‘Conventional shoeing protocols provide horses comfort during and after shoeling’ is a too strong conclusion that cannot be drawn due to the setup of your study. Suggestion: ‘ANS activity responds differently in horses undergoing different shoeing methods, with greater HRV 30-120 minutes after hot shoeing compared to cold shoeing. More research is needed to substantiate whether these differences can be attributed to the fact that horses experience more comfort from hot shoeing compared to cold shoeing.’
