# Supplementary material for: Heart rate and heart rate variability in horses undergoing hot and cold shoeing
Source: PLoS One. 2024 Jun 6;19(6):e0305031. doi: 10.1371/journal.pone.0305031 (PMC11156413; doi:10.1371/journal.pone.0305031)
Supplement: Attachment — Submitted filename: Review Comments to the Author.docx [file pone.0305031.s009.docx]

**Heart rate and heart rate variability in horses undergoing hot and cold shoeing (PONE-D-23-24799R4)**

Dear reviewer

First of all, we appreciate your dedication to reviewing our work and providing valuable comments to improve this manuscript. We’ve addressed all the points raised by the reviewer, and the corrections and mentions are highlighted in green.

Best regards,

Corresponding author

**Reviewer #2:**

The authors have addressed the points made previously and the manuscript has improved greatly. A few points in the discussion still need attention. If these are addressed correctly I do feel that this paper may be published.

Line 299-314: This section addresses the methodology and seems more appropriate within the M&M section. If the authors feel that this should remain within the discussion, please move the section more to the beginning (after line 282) and make clear why it is relevant to discuss this point. For example explain whether there is a debate within literature how HRV should be measured reliably and start the alinea with explaining this.

Response to the reviewers

We’d like to thank the reviewer for this valuable suggestion. After careful reading and interpretation again, we decided to remove this section from the discussion part as it seemed to be redundant. The core context in this discussion part has already been mentioned in the M&M part, for example, the validation of using HRM devices in horses (page 5, lines 102-103). The application of automatic artefact and noise correction provided by the Kubios premium program (page 7, lines 135-145).

Line 328-333: This section is not a discussion but a repetition of the results, as you do not relate to the existing literature. As the discussion section is already quite elaborate and these points are not the main ones, my suggestion would be to just leave it out.

Response to the reviewers

We’ve removed this section from the discussion section accordingly.

Line 391-401: The first sentence in still too bold, change it into: 'The findings of the study seem to confirm subjective evidence...." for example. Then still this sentence is more a conclusion from the previous section. It does not make clear what the rest of the alinea is about. So start with the main point of this alinea, what would you like to discuss here? The relationship of HRV and lameness? Perhaps here you could relate to the point that measuring locomotion and/or behavioural parameters may provide relevant/additional information?

Response to the reviewers

We’d like to thank the reviewer for the suggestion. We’ve revised this section to address the study’s findings and further study of HRV modulation in horses during locomotion and exercise in athletic horses on page 18, lines 371-377.

Line 403-414: Generally I do appreciate that an alinea is added that discusses the limitations, but sometimes a bit more explanation is needed. For example line 406-408: The sentence is not formulated correctly and it is unclear, it starts with Since....but since what? In addtition line 411 suffers from the same issue, explain why/how including behavioural parameters may render relevant or addition information that may confirm stress for example and clarify HRV differences. LIne 412-414, explain why inter-individual differences is an issue. Probably you mean that this may cause a bias as there may be influences of the locations on differences between hot/cold shoed groups?

Response to the reviewers

We’d like to thank the reviewer for the suggestion. We’ve revised the limitation of the study section to address the reviewer's concern on page 18-19, lines 378-390.
